# Risk Factors for Psychosis in the Migrant Population in Morocco

**DOI:** 10.1192/j.eurpsy.2025.1836

**Published:** 2025-08-26

**Authors:** N. Kissa, N. Ait Bensaid, A. Korchi, F. Laboudi, A. Ouanass

**Affiliations:** 1 University Psychiatric Hospital Arrazi, salé, Morocco

## Abstract

**Introduction:**

Immigration has been pivotal in shaping societies, especially post-war, but is associated with a 2 to 3 times higher risk of psychotic disorders for migrants compared to natives [5]. Psychotic disorders, affecting about 0.7% of the population, often involve chronic symptoms with high morbidity and mortality [1][3]. This increased risk in migrants highlights public health issues, particularly due to limited access to care [6][7]. Studies suggest that reducing specific ethnic risk factors could lower psychosis rates by 22% [8].

**Objectives:**

To identify risk factors and explanatory hypotheses for the development and symptomatic reactivation of psychoses in immigrant patients who were hospitalized at the Arrazi Psychiatric Hospital in Salé.

**Methods:**

This is a retrospective, descriptive, and analytical study. Data will be collected from the medical records of immigrant patients hospitalized for psychotic disorders at the Arrazi Psychiatric Hospital in Salé using a data extraction form. This form will include socio-demographic data, primary reason for migration, migration-related stress, social and professional situation, and access to mental health care.

**Results:**

Approximately 60% of immigrant patients hospitalized for psychotic disorders reported migration-related stress as a significant contributing factor. Around 45% experienced social isolation upon arrival, and 50% faced difficulties accessing mental health services. Additionally, 30% of these patients reported unemployment or precarious job situations, and nearly 40% cited family separation as a source of emotional strain, potentially exacerbating their symptoms.

**Conclusions:**

Psychosis in immigrants is often influenced by specific stress factors related to the migration experience, such as social isolation, economic insecurity, and limited access to mental health care. These elements increase the psychological vulnerability of migrants and can trigger or exacerbate psychotic disorders. Tailored support and policy measures are essential to mitigate these risks. These observations highlight the need for in-depth research to better understand and manage the risk factors for psychosis in this population.

**Disclosure of Interest:**

None Declared

